# An Analytical Model of Motion Artifacts in a Measured Arterial Pulse Signal—Part II: Tactile Sensors

**DOI:** 10.3390/s25185700

**Published:** 2025-09-12

**Authors:** Md Mahfuzur Rahman, Subodh Toraskar, Mamun Hasan, Zhili Hao

**Affiliations:** Department of Mechanical and Aerospace Engineering, Old Dominion University, Norfolk, VA 23529, USA; mrahm009@odu.edu (M.M.R.); stora002@odu.edu (S.T.); mhasa004@odu.edu (M.H.)

**Keywords:** arterial pulse measurement, baseline drift, dynamic systems, motion artifacts, time-varying system parameters (TVSP), TCS stack, tactile sensors

## Abstract

This paper, the second of two parts, presents an analytical model of motion artifacts (MA) in measured pulse signals by a tactile sensor, which contains a deformable microstructure sitting on a substrate. While the tissue-contact-sensor (TCS) stack and the sensor are both treated as a 1DOF (degree-of-freedom) system, tissue–sensor contact joins their mass together to form a 1DOF system with springs and dampers on both sides. MA on the sensor substrate causes baseline drift and time-varying system parameters (TVSP) of the TCS stack simultaneously. An analytical model is developed to mathematically relate baseline drift and TVSP to a measured pulse signal. The numerical calculation is conducted in MATLAB. Baseline drift in a measured pulse signal is much lower than the actual MA in its measurement. As compared to baseline drift, TVSP generates relatively abrupt, small distortion (e.g., 0.2% variation in heart rate and <5% change in pulse amplitude), but it rides on each harmonic of the true pulse signal. Sensor design alters both the deviation of the amplitude and waveform of a measured pulse signal from the true pulse signal and the influence of MA on it.

## 1. Introduction

Owing to their small size and low cost, various tactile sensors based on micro/nano-fabrication technologies have been developed for their vast potential applications [1]. Among them, arterial pulse measurement is a popular application targeted by tactile sensors [2,3,4,5,6,7,8,9,10,11,12]. These tactile sensors may take different forms with their unique features for certain advantages associated with pulse measurement [4,5,6,7,8,9,10,11,12]. Yet, in essence, these tactile sensors comprise of a deformable microstructure and a transducer sitting on a substrate [4,13]. In a measurement, a tactile sensor is placed on an artery. The true pulse signal in an artery passes through the transmission path: tissue, tissue–sensor contact, and the sensor, where the microstructure deforms in response to the input pulse signal. This deformation is recorded by the transducer. Thus, a tactile sensor measures the pulse signal as a displacement.

While photoplethysmography (PPG) sensors are mostly used at the index finger and accelerometers are mostly used at the carotid artery (CA), tactile sensors have been widely used at the radial artery (RA) and the CA [13]. As compared to a PPG sensor and an accelerometer, the deformable microstructure in a tactile sensor is involved in the transmission path [13], leading to increased complexity in analyzing the influence of motion artifacts (MA) on a measured pulse signal.

In this paper, we continue to treat the transmission path from the true pulse signal in an artery to the measured pulse signal by a sensor: tissue-contact-sensor (TCS) stack, as a 1DOF (degree-of-freedom) system. Although a tactile sensor itself, specifically its microstructure, is essentially a 1DOF system [13], tissue–sensor contact joins the mass from the microstructure and the mass of the overlying tissue together, leading to the formation of a 1DOF system with springs and dampers on both sides of its mass. For simplicity, the transducer is not included, and thus, the output of a tactile sensor is the displacement of the microstructure. Here, we follow the same methodology in Part I of the work to develop an analytical model for the influence of MA on a measured pulse signal by a tactile sensor.

## 2. Materials and Methods

The analytical model of MA is based on the same assumptions in Part I of this work and is not repeated here. For a better understanding of the work presented here, the readers are advised to read Part I of this work prior to this paper.

### 2.1. Arterial Wall Displacement as the True Pulse Signal

**Figure 1:**
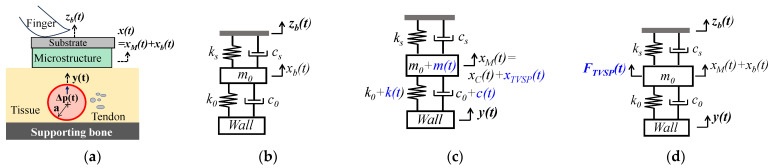
Schematics with arterial wall displacement *y*(*t*) as true pulse signal in an artery: (**a**) pulse measurement with a tactile sensor fixed via fingers; (**b**) 1DOF system of the TCS stack and the sensor with *z_b_*(*t*) at the sensor substrate; (**c**) 1DOF system with the TCS stack containing TVSP; (**d**) equivalent force for MA acting on the 1DOF system without TVSP (Note: TVSP-related terms are in blue).

As shown in Figure 1a,b, a tactile sensor is held at its substrate by fingers and is pressed against an artery with contact pressure *P_c_* to form the TCS stack, which is modeled as a 1DOF system. *P_c_* presets the nominal values of the TCS stack as *m*_0_, *k*_0_, and *c*_0_ and causes a static displacement in the sensor. This static displacement is not shown here and is excluded from the analysis, since its effect on a measured pulse signal is fully accounted for by the preset nominal values. With its substrate being fixed, the tactile sensor itself can be treated as a 1DOF system [13] with its own spring stiffness *k_s_* and damping coefficient *c_s_* from its microstructure. The sensor and the TCS stack join their mass together due to tissue–sensor contact, and thus *m*_0_ contains the contribution from the sensor and the tissue.

The TCS stack and the sensor form a 1DOF system with springs and dampers on both sides. As the true pulse signal, arterial wall displacement y(t)  serves as the base excitation for the 1DOF system and is time-harmonic [14,15,16]:

(1) $$y\left(t\right)\;=\;y_{0}e^{j{(\omega }_{y}t+\varphi_{y})}$$

where $y_{0}$, $\varphi_{y}$, and $\omega_{y}$ are the amplitude, phase, and angular frequency of y(t), respectively.

#### 2.1.1. MA as Baseline Drift and TVSP

As shown in Figure 1b, MA causes a time-varying displacement $z_{b}(t)$ at the sensor substrate:

(2) $$z_{b}\left(t\right)=\;z_{b0}e^{j{(\omega }_{b}t+\varphi_{b})}$$

where $z_{b0}$, $\varphi_{b}$, and $\omega_{b}$ are the amplitude, phase, and angular frequency of $z_{b}(t)$, respectively. This displacement serves as the base excitation for the 1DOF system and leads to the displacement $x_{b}(t)$ at the mass [13]:

(3) $$m_{0}\frac{d^{2}x_{b}\left(t\right)}{{dt}^{2}}+\left(c_{0}+c_{s}\right)\frac{dx_{b}\left(t\right)}{dt}+\left(k_{0}+k_{s}\right)\cdot x_{b}\left(t\right)=k_{s}z_{b}\left(t\right)+c_{s}\frac{dz_{b}\left(t\right)}{dt}$$

The sensor measures the relative distance of its substrate to the mass. Thus, the measured baseline drift by the sensor is $x_{b}\left(t\right)-{\;z}_{b}(t)$. It is $x_{b}(t)$ that causes the time-varying system parameters (TVSP) of the TCS stack:

(4) $$m=m_{0}+m(t),\;c=c_{0}+c(t),\;k=k_{0}+k\left(t\right)\mathrm{with}\;m\left(t\right),\;k(t),\;c(t)\propto x_{b}(t)$$

As shown in Figure 1c, y(t), as the base excitation, causes displacement $x_{M}(t)$ at the mass:

(5) $$\left[m_{0}+m\left(t\right)\right]\cdot \frac{d^{2}x_{M}\left(t\right)}{{dt}^{2}}+\left[c_{0}+c\left(t\right)+c_{s}\right]\cdot \frac{dx_{M}\left(t\right)}{dt}+\left[k_{0}+k\left(t\right)+k_{s}\right]\cdot x_{M}\left(t\right)=\left[k_{0}+k\left(t\right)\right]\cdot y\left(t\right)+\left[c_{0}+c(t)\right]\cdot \frac{dy\left(t\right)}{dt}$$

Due to TVSP in (5), $x_{M}(t)$ takes the form [17,18,19,20]:

(6) $${x_{M}(t)=x}_{T}(t)e^{j\varphi_{T}\left(t\right)}\;\mathrm{with}\;\omega_{T}(t)=\frac{{d\varphi }_{T}(t)}{dt}$$

where $x_{T}$, $\varphi_{T}$, and $\omega_{T}$ are the instantaneous amplitude, phase, and frequency of $x_{M}(t)$, respectively. The measured pulse signal by a tactile sensor $x_{tactile}\left(t\right)\;$ becomes:

(7) $$x_{tactile}\left(t\right)=x_{M}\left(t\right)+x_{b}\left(t\right)-\;z_{b}(t)\mathrm{with}\;x_{M}\left(t\right)=x_{C}\left(t\right)+x_{TVSP}(t)$$

where $x_{b}\left(t\right)-z_{b}(t)$ is the baseline drift; $x_{C}\left(t\right)$ is the measured pulse signal when free of MA (i.e., free of TVSP); and $x_{TVSP}(t)$ is the TVSP-generated distortion in Equation (5).

When the measurement is free of MA, based on Equation (5), the measured pulse signal $x_{C}\left(t\right)\;$ becomes [13]:

(8a) $$x_{C}\left(t\right)=x_{0}e^{j{(\omega }_{x}t+\varphi_{x})}=G_{0}e^{j\varphi_{0}}y_{0}e^{j{(\omega }_{y}t+\varphi_{y})}$$

(8b) $$\mathrm{with}\;G_{0}e^{j\varphi_{0}}\;=\frac{k_{0}+c_{0}j\omega_{y}}{\left[{-m}_{0}\omega_{y}^{2}+\left(c_{0}+c_{s}\right)j\omega_{y}+k_{0}+k_{s}\right]}\;\omega_{x}=\omega_{y};\;\varphi_{x}=\varphi_{y}+\varphi_{0};\;x_{0}=G_{0}y_{0}$$

Based on Equations (7), (8a) and (8b), the total distortion caused by MA in a measured pulse signal becomes:

(9) $$x_{tactile-MA}\left(t\right)=x_{TVSP}\left(t\right)+x_{b}\left(t\right)-{\;z}_{b}(t)\mathrm{with}\;x_{TVSP}\left(t\right)=x_{M}\left(t\right)\;-\;x_{C}\left(t\right)$$

#### 2.1.2. MA as Equivalent Forces

With the parameters of the TCS stack unaltered, the influence of MA on a measured pulse signal is accounted for by equivalent forces acting on the 1DOF system, as shown in Figure 1d. The 1DOF system is subject to three inputs: base excitation *y*(*t*) at the arterial wall, base excitation *z_b_*(*t*)at the sensor substrate, and *F_MA_*(*t*) on the mass [13]:

(10a) $$m_{0}\frac{d^{2}x\left(t\right)}{{dt}^{2}}+\left(c_{0}+c_{s}\right)\frac{dx\left(t\right)}{dt}+\left(k_{0}+k_{s}\right)\cdot x\left(t\right)=F_{MA}\left(t\right)+F_{b}\left(t\right)+F_{C}\left(t\right)$$

where

(10b) $$F_{C}\left(t\right)=k_{0}\cdot y\left(t\right)+c_{0}\cdot \frac{dy\left(t\right)}{dt}(Arterial wall)$$

(10c) $$F_{b}(t)=k_{s}z_{b}\left(t\right)+c_{s}\frac{dz_{b}\left(t\right)}{dt}(sensor substrate)$$

where $F_{C}\left(t\right)$ and $F_{b}\left(t\right)$ are the equivalent forces acting on the mass from y(t) and $z_{b}\left(t\right)$, respectively. The response of the 1DOF system to y(t) is identical to Equations (8a) and (8b). Equation (10a) should lead to the same displacement in Equation (7), and thus $F_{MA}\left(t\right)$ becomes:

(11a) $$F_{MA}\left(t\right)=F_{TVSP}\left(t\right)=F_{T}{\left(t\right)\;-\;F}_{C}\left(t\right)$$

where:

(11b) $$F_{T}\left(t\right)=m_{0}{\cdot \left(x_{T}\left(t\right)e^{j\varphi_{T}\left(t\right)}\right)}^{\prime \prime }+\left(c_{0}+c_{s}\right)\cdot {\left(x_{T}(t)e^{j\varphi_{T}\left(t\right)}\right)}^{\prime }+\left(k_{0}+k_{s}\right){\cdot x}_{T}(t)e^{j\varphi_{T}\left(t\right)}$$

Thus, the influence of MA on a measured pulse signal can be accounted for by $F_{TVSP}\left(t\right)$ acting on the mass and $z_{b}\left(t\right)$ at the sensor substrate. Because a tactile sensor detects the displacement difference between the mass and the sensor substrate, $z_{b}\left(t\right)$ cannot be simply replaced by $F_{b}\left(t\right)$ on the mass, as in the case of a PPG sensor and an accelerometer in Part I.

### 2.2. Arterial Pulsatile Pressure as the True Pulse Signal

**Figure 2:**
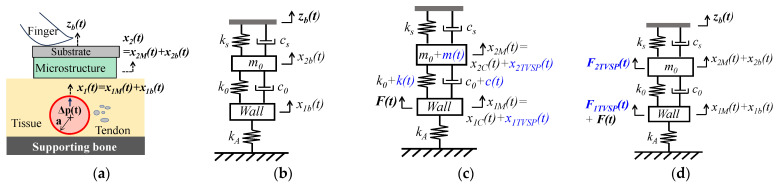
Schematics with pulsatile pressure Δ*p*(*t*) (i.e., *F*(*t*)) as true pulse signal in an artery: (**a**) pulse measurement with a tactile sensor fixed via fingers; (**b**) 2DOF system of the arterial wall, the TCS stack, and the tactile sensor with *z_b_*(*t*) at the sensor substrate; (**c**) 2DOF system with the TCS stack containing TVSP; (**d**) equivalent forces for MA acting on the 2DOF system without TVSP (Note: TVSP-related terms are in blue).

As shown in Figure 2a,b, the arterial wall is modeled as a spring with its stiffness *k_A_*. While one end of the spring is fixed, its other end is connected to the TCS stack. The addition of the arterial wall to the TCS stack forms a 2DOF system. Pulsatile pressure Δp(t)  is considered the true pulse signal, and translates to a force acting on the arterial wall [13]:

(12) $$\left(t\right)=F_{0}e^{j{(\omega }_{p}t+\varphi_{p})}=\pi a\Delta p\left(t\right)\;\mathrm{with}\;\Delta p\left(t\right)={\Delta p}_{0}e^{j{(\omega }_{p}t+\varphi_{p})}$$

where ${\Delta p}_{0}$, $\varphi_{p}$, and $\omega_{p}$ denotes the amplitude, phase, and angular frequency of Δp(t), respectively.

#### 2.2.1. MA as Baseline Drift and TVSP

As shown in Figure 2b, MA causes a time-varying displacement $z_{b}\left(t\right)$ at the sensor substrate:

(13) $$z_{b}(t)=z_{b0}e^{j{(\omega }_{b}t+\varphi_{b})}$$

This displacement leads to time-varying displacements $x_{1b}\left(t\right)$ and $x_{2b}\left(t\right)$ at the arterial wall and the mass, respectively [13]:

(14a) $$\left(k_{A}+k_{0}\right)x_{1b}\left(t\right)-\;k_{0}x_{2b}\left(t\right)+c_{0}\left[\frac{{dx}_{1b}\left(t\right)}{dt}\;-\;\frac{{dx}_{2b}\left(t\right)}{dt}\right]=0$$

(14b) $${-k_{0}x}_{1b}\left(t\right)-{\;c}_{0}\frac{{dx}_{1b}\left(t\right)}{dt}+m_{0}\frac{{d^{2}x}_{2b}\left(t\right)}{{dt}^{2}}+{{(k}_{0}+k_{s})x}_{2b}\left(t\right)+{(c}_{0}+c_{s})\frac{{dx}_{2b}\left(t\right)}{dt}=k_{s}z_{b}\left(t\right)+c_{s}\frac{dz_{b}\left(t\right)}{dt}$$

where the right side of Equation (14b) can be seen as base excitation at the sensor substrate:

(15) $$F_{b}(t)=k_{s}z_{b}\left(t\right)+c_{s}\frac{dz_{b}\left(t\right)}{dt}$$

Consequently, TVSP of the TCS stack in (4) are functions of $x_{2b}\left(t\right)\;-\;x_{1b}\left(t\right)$:

(16) $$k\left(t\right),\;c\left(t\right),\;m\left(t\right)\propto x_{2b}\left(t\right)-\;x_{1b}\left(t\right)$$

As shown in Figure 2c, the displacements at the mass and the wall are governed by [13]:

(17a) $$\left(k_{A}+k_{0}+k\left(t\right)\right){\cdot x}_{1M}\left(t\right)\;-\;\left(k_{0}+k\left(t\right)\right)\cdot x_{2M}\left(t\right)+(c_{0}+c\left(t\right))\cdot \left[\frac{{dx}_{1M}\left(t\right)}{dt}\;-\;\frac{{dx}_{2M}\left(t\right)}{dt}\right]=F\left(t\right)$$

(17b) $${-\left(k_{0}+k\left(t\right)\right)x}_{1M}\left(t\right)-\left(c_{0}+c\left(t\right)\right)\frac{{dx}_{1M}\left(t\right)}{dt}+\left(m_{0}+m\left(t\right)\right)\frac{{d^{2}x}_{2M}\left(t\right)}{{dt}^{2}}+{{(k}_{0}+k(t)+k_{s})x}_{2M}\left(t\right)+{(c}_{0}+c(t)+c_{s})\frac{{dx}_{2M}\left(t\right)}{dt}=0$$

The solution to $x_{1M}\left(t\right)$ and $x_{2M}\left(t\right)$ takes the forms [17,18,19,20]:

(18a) $$x_{1M}\left(t\right)=x_{1T}{(t)e}^{j\varphi_{1T}(t)}\;\mathrm{with}\;\omega_{1T}(t)=\frac{{d\varphi }_{1T}(t)}{dt}$$

(18b) $$x_{2M}\left(t\right)=x_{2T}{(t)e}^{j\varphi_{2T}(t)}\;\mathrm{with}\;\omega_{2T}(t)=\frac{{d\varphi }_{T2}(t)}{dt}$$

where $x_{1T}$, $\varphi_{1T}$, and $\varphi_{1T}$ are the instantaneous amplitude, phase, and frequency of $x_{1M}\left(t\right)$, respectively; and $x_{2T}$, $\varphi_{2T}$, and $\varphi_{2T}$ are the instantaneous amplitude, phase, and frequency of $x_{2M}\left(t\right),$ respectively. The measured pulse signal by the sensor $x_{tactile}\left(t\right)$ and the wall displacement $x_{wall}\left(t\right)\;$ become:

(19a) $$x_{tactile}\left(t\right)=x_{2M}\left(t\right)+x_{2b}\left(t\right)\;-\;z_{b}\left(t\right)\mathrm{with}\;x_{2M}\left(t\right)=x_{2C}\left(t\right)+x_{2TVSP}\left(t\right)$$

(19b) $$x_{wall}\left(t\right)=x_{1M}\left(t\right)+x_{1b}\left(t\right)\;\mathrm{with}\;x_{1M}\left(t\right)=x_{1C}\left(t\right)+x_{1TVSP}\left(t\right)$$

where *x_b_*(*t*) − *z_b_*(*t*) is the baseline drift in $x_{tactile}\left(t\right)$; $x_{2C}\left(t\right)$ is the measured pulse signal using a tactile sensor when free of MA (i.e., free of TVSP); and $x_{2TVSP}(t)$ is the TVSP-generated distortion at the mass in Equations (17a) and (17b). Similarly, $x_{1b}\left(t\right)$ is the baseline drift at the wall and $x_{1TVSP}\left(t\right)$ is the TVSP-generated distortion at the wall in Equations (17a) and (17b).

When free of MA, based on Equations (17a) and (17b), the displacement $x_{2C}(t)$ at the mass and the displacement $x_{1C}(t)$ at the arterial wall are [13]:

(20a) $$x_{1C}\left(t\right)=x_{10}e^{j{(\omega }_{x1}t+\varphi_{x1})}=G_{10}e^{j\varphi_{10}}F_{0}e^{j{(\omega }_{p}t+\varphi_{p})}$$

(20b) $$\mathrm{With}\;G_{10}e^{j\varphi_{10}}=\frac{1}{k_{A}-\frac{\left(c_{0}\omega_{p}j+k_{0})(m_{0}\omega_{p}^{2}-c_{s}\omega_{p}j-{\;k}_{s}\right)}{-m_{0}\omega_{p}^{2}+\left[c_{0}+c_{s}\right]\omega_{p}j+\left[k_{0}+k_{s}\right]}};\;\;\omega_{x1}=\omega_{p};{\;\varphi }_{x1}=\varphi_{p}+\varphi_{10};\;x_{10}=G_{10}F_{0}$$

(20c) $$x_{2C}\left(t\right)=x_{20}e^{j{(\omega }_{x2}t+\varphi_{x2})}=G_{20}e^{j\varphi_{20}}F_{0}e^{j{(\omega }_{p}t+\varphi_{p})}$$

(20d) $$\mathrm{With}\;G_{20}e^{j\varphi_{20}}=\frac{1}{k_{A}\left\{1+\frac{-{m_{0}\omega }_{p}^{2}+k_{s}+c_{s}\omega_{p}j}{c_{0}\omega_{p}j+k_{0}}\right\}-{m_{0}\omega }_{p}^{2}+k_{s}+c_{s}\omega_{p}j};\;\omega_{x2}=\omega_{p};\;\varphi_{x2}=\varphi_{p}+\varphi_{20};\;x_{20}=G_{20}F_{0}$$

Consequently, the measured pulse signal by the sensor is $x_{2C}\left(t\right)$. As compared to the displacements free of MA, the total distortion caused by MA at the mass (tactile sensor) $x_{tactile-MA}\left(t\right)$ and the wall $x_{wall-MA}\left(t\right)$ becomes:

(21a) $$x_{tactile-MA}\left(t\right)=x_{2TVSP}\left(t\right)+x_{2b}\left(t\right)-\;z_{b}\left(t\right)\;\mathrm{with}\;x_{2TVSP}\left(t\right)=x_{2M}\left(t\right)\;-\;x_{2C}\left(t\right)\;$$

(21b) $$x_{wall-MA}\left(t\right)=x_{1TVSP}\left(t\right)+x_{1b}(t)\;\mathrm{with}\;x_{1TVSP}\left(t\right)=x_{1M}\left(t\right)-x_{1C}\left(t\right)$$

#### 2.2.2. MA as Equivalent Forces

With the parameters of the TCS stack unaltered, we derive equivalent forces accounting for the influence of MA on the displacements at the wall and the sensor, as shown in Figure 2d. Based on Equations (19a) and (19b), equivalent forces should lead to the following displacements at the mass and the wall:

(22a) $$x_{2}\left(t\right)=x_{2M}\left(t\right)+x_{2b}\left(t\right)-\;x_{2C}\left(t\right)$$

(22b) $$x_{1}\left(t\right)=x_{1M}\left(t\right)+x_{1b}\left(t\right)-x_{1C}\left(t\right)$$

Then, $x_{1b}(t)$ and $x_{2b}(t)$ are caused by $z_{b}\left(t\right)$ at the sensor substrate. Based on Equations (17a) and (17b), the equivalent force associated with $x_{1C}\left(t\right)$ and $x_{2C}\left(t\right)$ should be −F(t) acting on the arterial wall. Equivalent forces associated with $x_{1M}\left(t\right)$ and $x_{2M}\left(t\right)$ are given by:

(23a) $${\left(k_{A}+k_{0}\right)x}_{1M}\left(t\right)+c_{0}\frac{{dx}_{1M}\left(t\right)}{dt}\;-\;k_{0}x_{2M}\left(t\right)\;-\;c_{0}\frac{{dx}_{2M}\left(t\right)}{dt}=F_{1T}\left(t\right)$$

(23b) $${-k_{0}x}_{1M}\left(t\right)-c_{0}\frac{{dx}_{1M}\left(t\right)}{dt}+m_{0}\frac{{d^{2}x}_{2M}\left(t\right)}{{dt}^{2}}+{{(k}_{0}+k_{s})x}_{2M}\left(t\right)+{(c}_{0}+c_{s})\frac{{dx}_{2M}\left(t\right)}{dt}=F_{2T}\left(t\right)$$

Taken together, the influence of MA on a measured pulse signal can be accounted for by $z_{b}(t)$ at the sensor substrate and $F_{1MA}\left(t\right)$ acting on the arterial wall and $F_{2MA}\left(t\right)$ acting on the mass:

(24a) $$F_{1MA}\left(t\right)=F_{1TVSP}\left(t\right)\;\mathrm{with}\;F_{1TVSP}\left(t\right)=F_{1T}\left(t\right)\;-\;F\left(t\right)$$

(24b) $$F_{2MA}\left(t\right)=F_{2TVSP}\left(t\right)\;\mathrm{with}\;F_{2TVSP}\left(t\right)=F_{2T}\left(t\right)$$

As such, it is the combination of $F_{1TVSP}\left(t\right)$ acting on the wall and $F_{2TVSP}\left(t\right)$ acting on the mass that results in the TVSP-generated distortion in Equations (17a) and (17b).

### 2.3. Numerical Calculation

**Figure 18:**
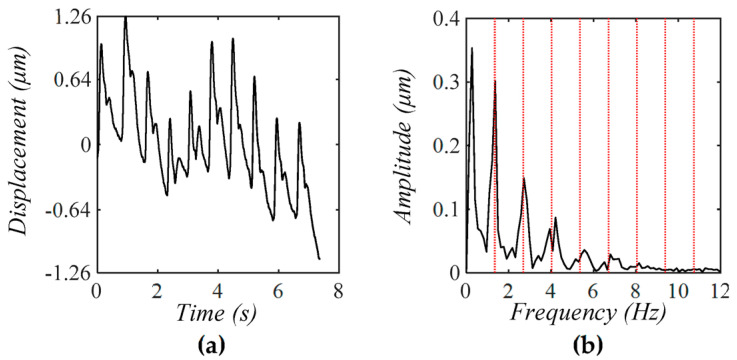
(**a**) A measured pulse signal at-rest at the CA using a tactile sensor [3] with a high level of MA and (**b**) its frequency spectrum (The redlines correspond to the frequency of the harmonics).

The same pulse signal and the same nominal values of *m*_0_, *k*_0_, and *c*_0_ in Part I are used here. Briefly, their nominal values are: $k_{0}=1/6\cdot k_{A}$, $r_{0}=\omega_{0}/\omega_{C}=2$, and $\zeta_{0}=1.5$, where $\omega_{0}$ and $\zeta_{0}$ are the nominal frequency ratio and damping factor of the TCS stack with $\omega_{C}$ being the frequency of the heart rate. The stiffness and damping factor of a tactile sensor are: $k_{s}=6\cdot k_{o}$ and $\zeta_{s}=1.5$. Displacement $z_{b}(t)$ at the sensor substrate (see Figure 18) and the relation of baseline drift to TVSP are assumed as the same in Part I. Then, measured pulse signals can be calculated using the model presented above. All the calculations are conducted in MATLAB2024a using the same functions as detailed in Part I.

## 3. Results

### 3.1. Arterial Wall Displacement as the True Pulse Signal

**Figure 3:**
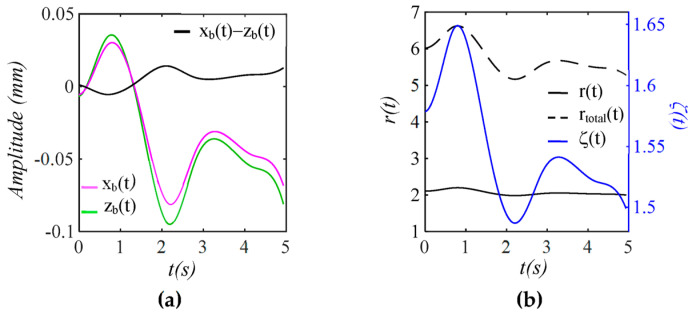
Baseline drift and TVSP in the TCS stack in Figure 1: (**a**) *x_b_*(*t*), *z_b_*(*t*), and *x_b_*(*t*) − *z_b_*(*t*); (**b**) *r*(*t*), *r_total_*(*t*), and ζ(*t*).

As shown in Figure 3, while $z_{b}(t)$ represents the actual MA, $x_{b}\left(t\right)$ is the baseline drift experienced by the TCS stack, which dictates TVSP. In contrast, the baseline drift in a measured pulse signal, $x_{b}\left(t\right)\;-\;z_{b}(t)$, is much smaller than the actual MA and is opposite to the changing trend of MA. Frequency ratio r(t) and damping factor ζ(t) of the TCS stack accounting for TVSP follow the changing trend of $x_{b}\left(t\right)$. Frequency ratio $r_{total}(t)$, which accounts for *k_s_*, shows that the sensor greatly increases the frequency of the 1DOF system.

**Figure 4:**
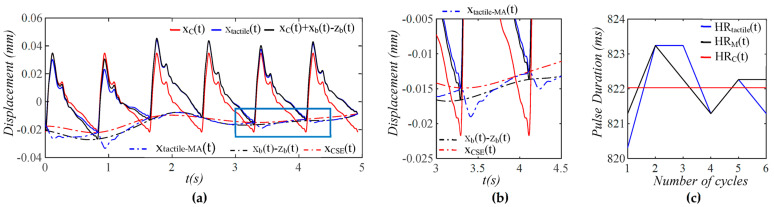
Calculated measurement at the mass (tactile sensor measurement) (**a**) pulse signals: *x_C_*(*t*), *x_tactile_*(*t*), and *x_C_*(*t*) + *x_b_*(*t*) − *z_b_*(*t*), MA-related signals: *x_b_*(*t*) − *z_b_*(*t*), *x_tactile-MA_*(*t*), and *x_CSE_*(*t*). (**b**) zoom-in view of MA-related signals (**c**) HR: *HR_tactile_*, *HR_M_*, and *HR_C_*.

As shown in Figure 4, $x_{tactile}\left(t\right)$ contains both TVSP-generated distortion $x_{TVSP}\left(t\right)$ and measured baseline drift $x_{b}\left(t\right)-z_{b}(t)$, and the pulse signal $x_{C}\left(t\right){+x}_{b}\left(t\right)-z_{b}(t)$ excludes $x_{TVSP}\left(t\right)$. As compared to $x_{b}\left(t\right)-z_{b}(t)$, $x_{TVSP}\left(t\right)$ is abrupt and relatively large. Due to $x_{TVSP}\left(t\right)$, $x_{b}\left(t\right)-z_{b}(t)$ is noticeably off the start/end of the pulse cycles in $x_{tactile}\left(t\right)$. In contrast, $x_{tactile-MA}\left(t\right)$, the sum of $x_{b}\left(t\right)-z_{b}(t)$ and $x_{TVSP}\left(t\right)$, matches the start/end of the pulse cycles in $x_{tactile}\left(t\right)$. The CSE-estimated baseline drift $x_{CSE}(t)$ is different from $x_{b}\left(t\right)-z_{b}(t)$. In Figure 4c, *HR_Tactile_* and *HR_M_* are heart rate (HR) derived from $x_{tactile}\left(t\right)$ and $x_{M}\left(t\right)$ (including only $x_{TVSP}\left(t\right)$), respectively. For comparison, *HR_C_* is HR derived from $x_{C}\left(t\right)$, which is identical to HR from y(t). Baseline drift and TVSP both shift the start/end of pulse cycles.

**Figure 5:**
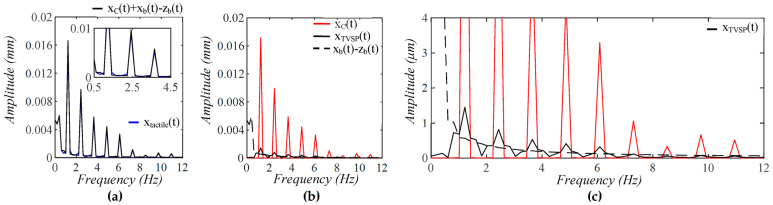
Frequency spectrum of (**a**) *x_tactile_*(*t*) and *x_C_*(*t*) + *x_b_*(*t*) − *z_b_*(*t*); (**b**) *x_C_*(*t*), *x_TVSP_*(*t*), and *x_b_*(*t*) − *z_b_*(*t*); (**c**) zoom-in view of *x_TVSP_*(*t*).

**Figure 6:**
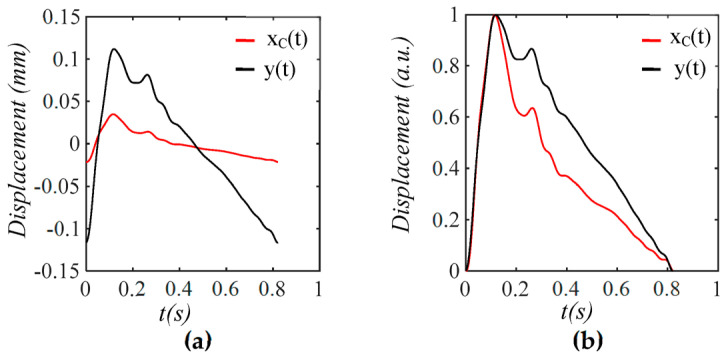
The amplitude and APW of the measured pulse signal free of MA *x_C_*(*t*) is different from the true pulse signal *y*(*t*) *= F*(*t*)/*k_A_* due to the harmonic-dependent transfer function of the TCS stack in Equations (8a) and (8b). (**a**) Pulse signals; (**b**) their normalized APW.

**Figure 7:**
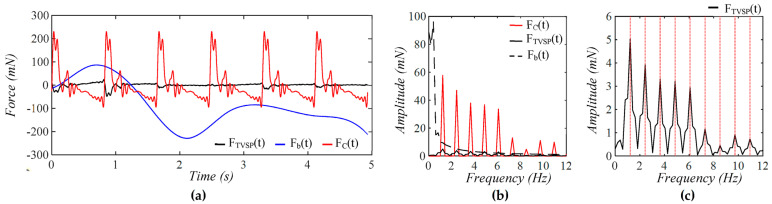
Calculated equivalent forces for MA: (**a**) *F_C_*(*t*), *F_TVSP_*(*t*), and *F_b_*(*t*); (**b**) frequency spectrum of *F_C_*(*t*), *F_TVSP_*(*t*), and *F_b_*(*t*); (**c**) frequency spectrum of *F_TVSP_*(*t*) (The redlines correspond to the frequency of harmonics).

As shown in Figure 5, each harmonic of $x_{C}\left(t\right)$ appears as a distinct, sharp peak. Baseline drift appears as a low-frequency peak. In contrast, TVSP causes low-amplitude signals centering around each harmonic of the true pulse signal. Because the lower harmonics in the true pulse signal are large, TVSP causes more distortion to the first~fifth harmonics. As shown in Figure 6, the measured pulse amplitude free of MA is much smaller than the true pulse amplitude, and the measured APW greatly deviates from the true APW. The difference between the two arises from the TCS stack and the tactile sensor, which work together as a harmonics-dependent transfer function between them, as shown in Equations (8a) and (8b). As shown in Figure 7, the equivalent force $F_{TVSP}\left(t\right)$ at the mass is very small relative to $F_{C}\left(t\right)$, but is large enough to cause observable distortion and HR variations in a measured pulse signal. $F_{TVSP}\left(t\right)$ has its signals centering around each harmonic of the true pulse signal. In contrast, $F_{b}(t)$ from the sensor substrate is comparable to $F_{C}\left(t\right)$ in terms of magnitude and contains a large low-frequency peak. It should be emphasized again that $F_{b}(t)$ represents the actual MA in a pulse measurement, and yet the baseline drift in a measured pulse signal fails to capture this high-level MA.

### 3.2. Pulsatile Pressure as the True Pulse Signal

**Figure 8:**
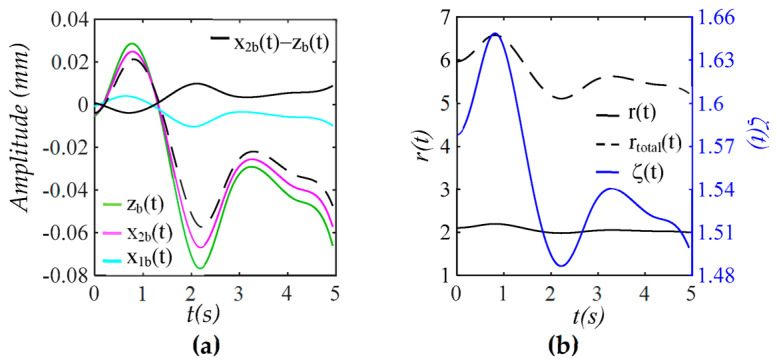
Baseline drift and TVSP of the TCS stack: (**a**) *z_b_*(*t*), *x*_2*b*_(*t*), and *x*_1*b*_(*t*), measured baseline drift *x*_2*b*_(*t*) − *z_b_*(*t*) and TVSP-related baseline drift *x*_2*b*_(*t*) − *x*_1*b*_(*t*); (**b**) *r*(*t*), *r_total_*(*t*), and *ζ*(*t*).

When Δp(t) (i.e., F(t)) is the true pulse signal, as shown in Figure 8, $z_{b}(t)$ represents the actual MA, which leads to a comparable $x_{2b}\left(t\right)$ but a small $x_{1b}\left(t\right)$. While the measured baseline drift by a sensor $x_{2b}\left(t\right)-z_{b}(t)$ is small, the TVSP-related baseline drift $x_{2b}\left(t\right)-x_{1b}(t)$ is relatively large but is still smaller than the actual MA. While r(t) and ζ(t) follow the changing trend of $x_{2b}\left(t\right)-x_{1b}(t)$, $r_{total}(t)$ accounting for *k_s_* and *k_A_* is greatly increased, as compared to r(t).

**Figure 9:**
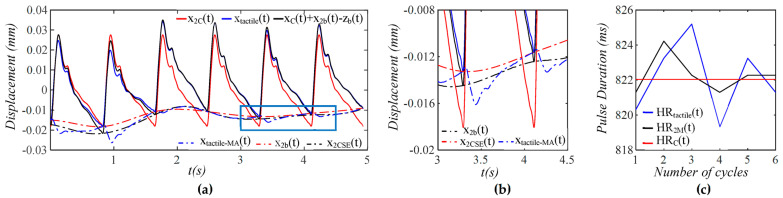
Calculated measurement at the mass (tactile sensor measurement) (**a**) pulse signals: *x*_2*C*_(*t*), *x_tactile_*(*t*), *x*_2*C*_(*t*) + *x*_2*b*_(*t*) − *z_b_*(*t*), MA-related signals: *x_tactile-MA_*(*t*), *x*_2*b*_(*t*) − *z_b_*(*t*), and *x*_2*CSE*_(*t*) (**b**) zoom-in view of MA-related signals (**c**) HR: *HR_tactile_*(*t*), *HR*_2*M*_(*t*), and *HR_C_*(*t*).

**Figure 10:**
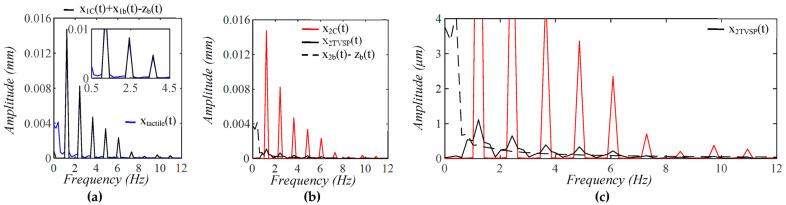
Frequency spectrum of (**a**) *x_tactile_*(*t*), *x*_2*C*_(*t*) + *x*_2*b*_(*t*) − *z_b_*(*t*); (**b**) *x*_2*C*_(*t*), *x*_2*TVSP*_(*t*), and *x*_2*b*_(*t*) − *z_b_*(*t*); (**c**) zoom-in view of *x*_2*TVSP*_(*t*).

As shown in Figure 9, as compared to baseline drift $x_{2b}\left(t\right)-z_{b}(t)$, TVSP-generated distortion $x_{2TVSP}\left(t\right)$ is quite abrupt and relatively large in the first two pulse cycles. This large $x_{2TVSP}\left(t\right)$ noticeably distorts the pulse waveform in these two pulse cycles. In Figure 9b, $x_{2TVSP}\left(t\right)$ and $x_{2b}\left(t\right)-z_{b}(t)$ both noticeably shift the start/end of the pulse cycles, relative to the start/end of $x_{2C}\left(t\right)$. In Figure 9c, *HR_Tactile_* and *HR*_2*M*_ are *HR* derived from $x_{tactile}\left(t\right)$ (including $x_{tactile-MA}\left(t\right)$) and $x_{2M}\left(t\right)$ (containing only $x_{2TVSP}\left(t\right)$), respectively. For comparison, *HR_C_* is HR derived from $x_{2C}\left(t\right)$, which is identical to HR from Δp(t). $x_{2TVSP}\left(t\right)$ and $x_{2b}\left(t\right)-z_{b}(t)$ alters HR to similar extents. As shown in Figure 10, while each harmonic of $x_{2C}\left(t\right)$ appears as a distinct, sharp peak, $x_{2b}\left(t\right)-z_{b}(t)$ has a low-frequency peak, and $x_{2TVSP}\left(t\right)$ has its signals centering around each harmonic of the true pulse signal.

**Figure 11:**
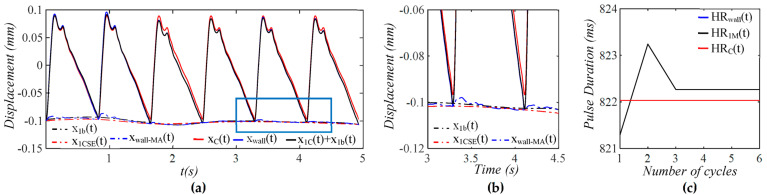
Calculated displacements at the arterial wall (**a**) pulse signals: *x*_1*C*_(*t*), *x_wall_*(*t*), *x*_1*C*_(*t*) + *x*_1*b*_(*t*), MA-related signals: *x_wall-MA_*(*t*), *x*_1*b*_(*t*), and *x*_1*CSE*_(*t*); (**b**) zoom-in view of MA-related signals; (**c**) HR variations: *HR_wall_*(*t*), *HR*_1*M*_(*t*), and *HR_C_*(*t*) (Note that the blue line is not shown, due to *HR_wall_*(*t*) = *HR_C_*(*t*)).

As shown in Figure 11, baseline drift at the arterial wall is $x_{1b}\left(t\right)$, and the arterial wall displacement $x_{wall}\left(t\right)$ contains $x_{1b}\left(t\right)$ and TVSP-generated distortion $x_{1TVSP}\left(t\right)$. Despite small $x_{1b}\left(t\right)$, $x_{1TVSP}\left(t\right)$ noticeably distorts the pulse waveform of the first two pulse cycles. The CSE-estimated baseline drift $x_{1CSE}(t)$ still differs from $x_{1b}\left(t\right)$. It is worth noting that $x_{wall}\left(t\right)$ encounters small TVSP-generated distortion, as compared to $x_{tactile}\left(t\right)$. In Figure 11c, *HR_wall_* and $x_{1M}\left(t\right)$ (containing only $x_{1TVSP}\left(t\right)$), respectively. It is the only $x_{1TVSP}\left(t\right)$ that causes HR variations between pulse cycles. In contrast, $x_{1b}\left(t\right)$ does not cause any HR variation, as compared to *HR*.

**Figure 12:**
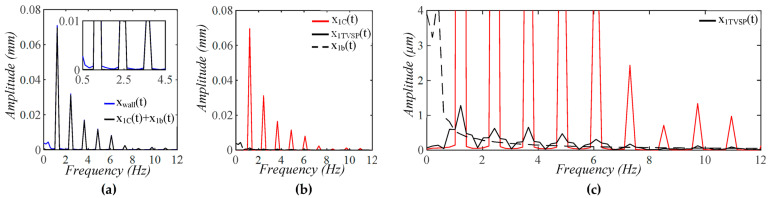
Frequency spectrum of (**a**) *x_wall_*(*t*), *x*_1*C*_(*t*) + *x*_1*b*_(*t*); (**b**) *x*_1*C*_(*t*), *x*_1*TVSP*_(*t*), *x*_1*b*_(*t*); (**c**) zoom-in view of *x*_1*TVSP*_(*t*).

**Figure 13:**
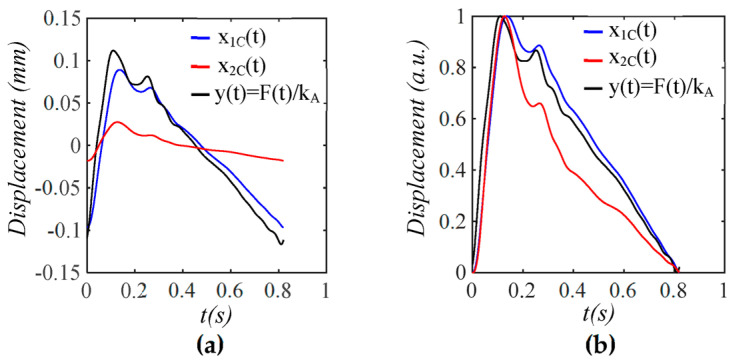
The amplitude and APW of measured pulse signal *x*_2*C*_(*t*) and wall displacement *x*_1*C*_(*t*) free of MA are different from the true pulse signal *y*(*t*) *= F*(*t*)/*k_A_* due to the harmonic-dependent transfer function of the TCS stack in Equations (20a)–(20d). (**a**) Pulse signals; (**b**) their normalized APW.

As shown in Figure 12, $x_{1TVSP}\left(t\right)$ has its signals centering around each harmonic of the true pulse signal. Baseline drift $x_{1b}\left(t\right)$ has an extremely small low-frequency peak. Figure 13 compares $x_{1C}\left(t\right)$, $x_{2C}\left(t\right)$, and $y(t)=F(t)/k_{A}\;$(free of measurement). The difference between $x_{1C}\left(t\right)$ (wall displacement) and y(t) stems from the interference of the TCS stack and the sensor with the arterial wall motion in response to Δp(t), as shown in Equations (20a)–(20d). Evidently, the arterial wall displacement is affected by pulse measurement to some extent. In contrast, the amplitude and APW of $x_{2C}\left(t\right)$ (measured pulse signal) significantly deviate from *y*(*t*).

**Figure 14:**
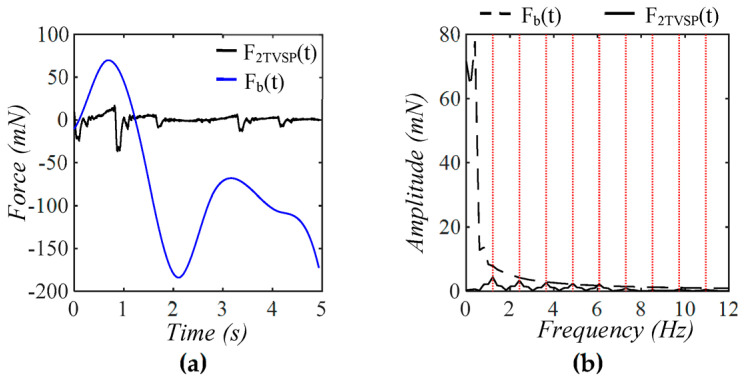
Calculated equivalent forces for MA acting on the mass: (**a**) *F*_2*TVSP*_(*t*) and *F_b_*(*t*); (**b**) frequency spectrum of *F*_2*TVSP*_(*t*) and *F_b_*(*t*) (The redlines correspond to the freuency of the harmonics).

**Figure 15:**
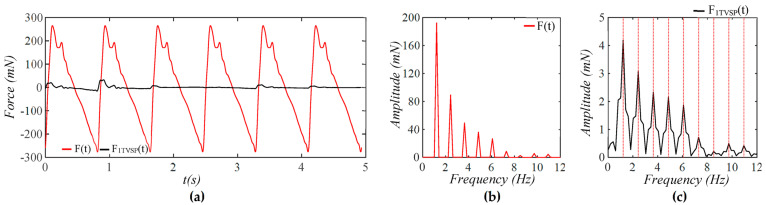
Calculated equivalent forces for MA acting on the arterial wall: (**a**) *F*(*t*) and *F*_1*TVSP*_(*t*); (**b**) frequency spectrum of *F*(*t*); (**c**) frequency spectrum of *F*_1*TVSP*_(*t*) (The redlines correspond to the frequencies of the harmonics).

As shown in Figure 14, $F_{2TVSP}\left(t\right)$ varies sort of randomly and abruptly over time, as compared to $F_{b}(t)$. While $F_{b}(t)$ appears as a quite large low-frequency peak, $F_{2TVSP}\left(t\right)$ has its signals centering around each harmonic of the true pulse signal. As shown in Figure 15, as compared to F(t), $F_{1TVSP}\left(t\right)$ is small and centers around each harmonic of F(t). A comparison of Figure 14 and Figure 15 with Figure 9 and Figure 11 reveals that the abrupt changes in $F_{1TVSP}\left(t\right)$ and $F_{2TVSP}\left(t\right)$ correspond to the abrupt changes in *x*_1*TVSP*_(*t*) and *x*_2*TVSP*_(*t*).

**Figure 16:**
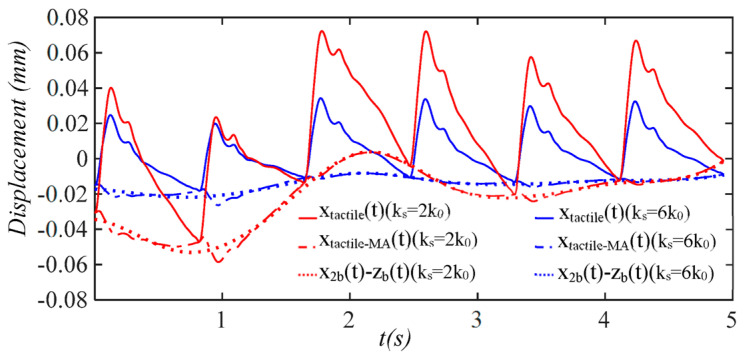
Calculated results at the mass (tactile sensor measurement): pulse signal *x_tactile_*(*t*) and MA-related signals; *x_tactile-MA_*(*t*), *x*_2*b*_(*t*) − *z_b_*(*t*) with sensor stiffness; *k_s_ = 6k*_0_ and *k_s_ = 2k*_0_.

**Figure 17:**
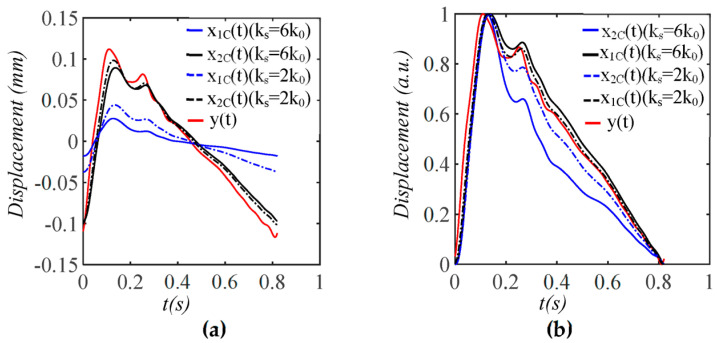
The effect of sensor stiffness on the measured pulse signal *x*_2*C*_(*t*) and the wall displacement *x*_1*C*_(*t*) when free of MA (note: *y*(*t*) = *F*(*t*)/*k_A_* is the true pulse signal). (**a**) Pulse signals; (**b**) their normalized APW.

Figure 16 shows the effect of the sensor stiffness *k_s_* on the influence of MA on a measured pulse signal. A low-stiffness sensor gives rise to a measured pulse signal with large amplitude but encounters high TVSP-generated distortion under the same level of MA. Yet, as shown in Figure 17, with a low-stiffness sensor, the true pulse signal is less affected, and the measured APW deviates from the true APW to a much lesser extent.

## 4. Discussion

The novelty of this study also lies in the full consideration of the involvement of the transmission path in pulse measurement, as compared with the existing studies on pulse measurement by various tactile sensors. The study limitations in this study are the same as those discussed in Part I and are not repeated here. Similarly, physical implications for arterial wall displacement and pulsatile pressure serving as the true pulse signal, as well as the unsuitability of the current data-processing algorithms for estimation of MA, remain the same and are not repeated. Here, we focus on discussing the implications of the results in Section 3 on the influence of MA on a measured pulse signal by a tactile sensor and comparing the difference between a tactile sensor and an accelerometer pertaining to pulse measurement.

### 4.1. Baseline Drift Versus TVSP-Generated Distortion

As shown in Section 3, $z_{b}\left(t\right)$ at the sensor substrate represents the level of MA in pulse measurement. Regardless of which signal is used as the true pulse signal, baseline drift in a measured pulse signal is greatly smaller than the actual MA; baseline drift experienced by the TCS stack is comparable to the actual MA. Since it is baseline drift experienced by the TCS stack that dictates TVSP, TVSP-generated distortion in a measured pulse signal is comparable to the baseline drift in a measured pulse signal in terms of magnitude. Both TVSP-generated distortion and the measured baseline drift cause HR variations to similar extents. While the measured baseline drift has a gradually changing trend, the TVSP-generated distortion is relatively abrupt. Baseline drift lies in a low-frequency range. In contrast, TVSP-generated distortion rides on each harmonic of the true pulse signal, making it difficult to remove it from the measured signal via wavelet-based filtering and Empirical Modulation Decomposition (EMD). Given the effect of baseline drift and TVSP-generated distortion on shifting the start/end of a pulse cycle, direct employment of CSE for estimating baseline drift may lead to large errors in the derived APW.

### 4.2. Existence of TVSP

**Figure 19:**
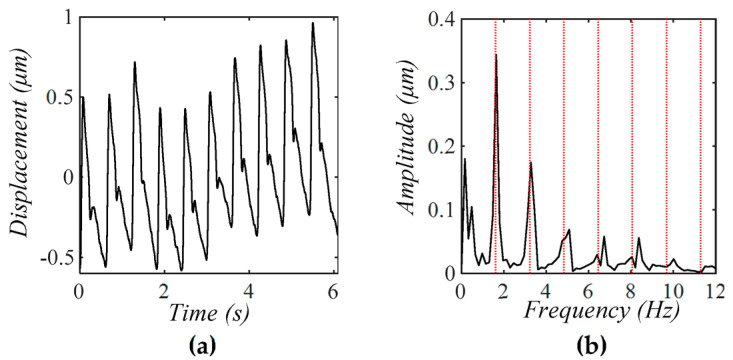
(**a**) A measured pulse signal 20 min post-exercise at the CA using a tactile sensor [3] with a low level of MA and (**b**) its frequency spectrum (The redlines correspond to the frequency of the harmonics).

To reveal the existence of TVSP in a measured pulse signal, Figure 18 and Figure 19 show two measured pulse signals at the CA by a tactile sensor on a 30 year-old healthy subject in a sitting position at two conditions: at rest and 20 min post-exercise, respectively (under the IRB approval at Old Dominion University) at our lab. The measured signal in Figure 18a is subject to a high level of MA (The actual MA might be even much higher, based on the results in Section 3), and thus, as expected, the measured amplitude and APW vary greatly between the pulse cycles. In particular, the fifth and sixth pulse cycles in Figure 18a suffer great distortion, which cannot be simply accounted for by the corresponding changing trend of baseline drift. As shown in Figure 18b, the peak at 0.104 Hz for baseline drift is even larger than the peak of the first harmonic of the measured pulse signal, indicating a high level of MA. Noticeably low-amplitude signals are centering around the first~fifth harmonics and are asymmetrical about each harmonic, testifying to the existence of TVSP.

The measured signal in Figure 19a suffers from a low level of MA, and thus, the measured amplitude and APW vary moderately between pulse cycles. As shown in Figure 19b, the baseline drift at 0.16 Hz and 0.49 Hz is smaller than the 1st harmonic of the measured pulse signal. Meanwhile, the low-amplitude signals centering around the first~fifth harmonics and being asymmetrical also testify to the existence of TVSP. Comparison of these lower-amplitude signals between Figure 18b and Figure 19b indicates that the TVSP-generated distortion in Figure 18b is larger than that in Figure 19b.

### 4.3. Comparison of a Tactile Sensor and an Accelerometer

Pulse signals at the CA are more indicative of the aortic condition [14,15,16], as compared to those at the index finger and the wrist. Tactile sensors and accelerometers are both used at the CA. In contrast, a PPG sensor is mostly used on the index finger [21]. Thus, we compare the difference only between a tactile sensor and an accelerometer. As shown in Figure 1 and Figure 2, manual fixing of a tactile sensor at an artery does not form part of the TCS stack, other than presetting its nominal values, compared with an accelerometer. However, compared with an accelerometer, the spring *k_s_* and damper *c_s_* of a tactile sensor contribute to the harmonic-dependent transfer function from the true pulse signal to the measured pulse signal. This contribution undermines the comparability between studies using different tactile sensors. In particular, the measured pulse signal is greatly influenced by the sensor design (e.g., *k_s_*), in terms of amplitude, APW, as well as the TVSP-generated distortion. A low-stiffness sensor gives rise to a measured pulse signal with a large amplitude and APW less deviated from the true one, but high baseline drift and high TVSP-generated distortion under the same level of MA.

Compared with an accelerometer, one significant advantage of a tactile sensor lies in the fact that it measures the displacement directly, and the measured displacement covers a large portion of its measurement range. As such, the measured pulse signal by a tactile sensor is much more immune to sensor noise and free of any accumulated noise from the integral process, as compared to its counterpart by an accelerometer [22,23]. Furthermore, manual fixing of a tactile sensor leads to a low level of MA, whereas fixing an accelerometer with tape is prone to a high level of MA (see Part I). Comparison of the measured pulse signals in Figure 18 and Figure 19 with their counterparts by an accelerometer in Part I clearly reveals these two great advantages offered by a tactile sensor. This may explain the reason why numerous studies exist on pulse measurement by various tactile sensors, but only a few studies have reported on pulse measurement by an accelerometer [13]. Practically speaking, a tactile sensor serves as a much better choice for pulse measurement than an accelerometer, in terms of the effect of MA and measured APW. It should be noted that an accelerometer offers its advantage in heart rate measurement over a tactile sensor [24,25,26].

## 5. Conclusions

An analytical model of MA in a measured pulse signal by a tactile sensor is presented here. MA causes baseline drift and TVSP of the TCS stack simultaneously. Baseline drift in a measured pulse signal is well below the actual MA encountered in its measurement. While baseline drift has a gradually changing trend, TVSP-generated distortion is relatively abrupt; it can cause 0.2% variation in heart rate, but more importantly, it rides on each harmonic of the true pulse signal and distorts the information of each harmonic in a measured pulse signal. In addition to affecting the deviation of the amplitude and APW of a measured pulse signal from the true pulse signal, sensor design can greatly alter the influence of MA on a measured pulse signal. Together with Part I, this study reveals the need to quantify the influence of the TCS stack, MA, and sensor design on a measured pulse signal for improving measurement accuracy or reliability. The analytical model presented here may serve as a fundamental framework for quantifying such influence in the future. Given the six parameters in the TCS stack and baseline drift, time-frequency analysis on a measured pulse signal is needed to truly quantify these parameters and baseline drift to accurately estimate MA and the deviation from the TCS stack and ultimately attain the true pulse signal.

## Data Availability

The data that support the findings of this study are available upon reasonable request from the corresponding author. The data are not publicly available due to privacy or ethical restrictions.

## Abbreviations

The following abbreviations are used in this manuscript:

BD	Baseline drift
DOF	Degree-of-freedom
TVSP	Time-varying system parameters
MA	Motion artifacts
TCS	Tissue-contact-sensor
HR	Heart rate
